# Mutation intolerant genes and targets of FMRP are enriched for nonsynonymous alleles in schizophrenia

**DOI:** 10.1002/ajmg.b.32560

**Published:** 2017-07-18

**Authors:** Ganna Leonenko, Alexander L. Richards, James T. Walters, Andrew Pocklington, Kimberly Chambert, Mariam M. Al Eissa, Sally I. Sharp, Niamh L. O'Brien, David Curtis, Nicholas J. Bass, Andrew McQuillin, Christina Hultman, Jennifer L. Moran, Steven A. McCarroll, Pamela Sklar, Benjamin M. Neale, Peter A. Holmans, Michael J. Owen, Patrick F. Sullivan, Michael C. O'Donovan

**Affiliations:** ^1^ Division of Psychological Medicine and Clinical Neurosciences MRC Centre for Neuropsychiatric Genetics and Genomics, Cardiff University School of Medicine Cardiff UK; ^2^ Stanley Center for Psychiatric Research Broad Institute of MIT and Harvard Cambridge Massachusetts; ^3^ Division of Psychiatry, Molecular Psychiatry Laboratory University College London London UK; ^4^ UCL Genetics Institute, UCL London UK; ^5^ Department of Medical Epidemiology and Biostatistics Karolinska Institute Stockholm Sweden; ^6^ Program in Medical and Population Genetics Broad Institute of MIT and Harvard Cambridge Massachusetts; ^7^ Department of Genetics Harvard Medical School Boston Massachusetts; ^8^ Icahn School of Medicine at Mount Sinai New York New York; ^9^ Analytical and Translational Genetics Unit Massachusetts General Hospital Boston Massachusetts; ^10^ Departments of Genetics and Psychiatry University of North Carolina Chapel Hill North Carolina

**Keywords:** association, exome chip, FMRP, rare variation, schizophrenia

## Abstract

Risk of schizophrenia is conferred by alleles occurring across the full spectrum of frequencies from common SNPs of weak effect through to ultra rare alleles, some of which may be moderately to highly penetrant. Previous studies have suggested that some of the risk of schizophrenia is attributable to uncommon alleles represented on Illumina exome arrays. Here, we present the largest study of exomic variation in schizophrenia to date, using samples from the United Kingdom and Sweden (10,011 schizophrenia cases and 13,791 controls). Single variants, genes, and gene sets were analyzed for association with schizophrenia. No single variant or gene reached genome‐wide significance. Among candidate gene sets, we found significant enrichment for rare alleles (minor allele frequency [MAF] < 0.001) in genes intolerant of loss‐of‐function (LoF) variation and in genes whose messenger RNAs bind to fragile X mental retardation protein (FMRP). We further delineate the genetic architecture of schizophrenia by excluding a role for uncommon exomic variants (0.01 ≤ MAF ≥ 0.001) that confer a relatively large effect (odds ratio [OR] > 4). We also show risk alleles within this frequency range exist, but confer smaller effects and should be identified by larger studies.

## 1 INTRODUCTION

Schizophrenia is a highly heritable disorder with an average lifetime risk of 0.5–1%, although this can vary across and within countries (Gottesman & Shields, 1967). Prior studies point to a multifactorial aetiology involving genetic and environmental factors and an overall heritability of around 65% (Cardno & Gottesman, 2000; Lichtenstein et al., 2009; Sullivan, Kendler, & Neale, 2003). Genomic studies have decisively supported work from the pre‐molecular era suggesting that schizophrenia is highly polygenic and it is now clear that the large number of risk alleles involved span the full spectrum of frequencies from common through rare including de novo mutations (Purcell et al., 2009). The evidence to date from copy number variants (CNVs) supports the hypothesis that alleles that confer high risk of schizophrenia are subjected to strong selection pressure, but are maintained in the population at low frequencies by de novo mutation (Rees, Moskvina, Owen, O'Donovan, & Kirov, 2011) and incomplete penetrance. At the other end of the effect size spectrum, alleles conferring small effects on risk can become common.

Published genome‐wide association studies (GWAS) have identified over 100 genetic loci containing common alleles (The Psychiatric Genomics Consortium, 2014). Individually, common risk alleles contribute small effects (odds ratios typically <1.1) but en masse, it has been estimated half to a third of the genetic risk of schizophrenia is indexed by common alleles genotyped by current genome‐wide association study (GWAS) arrays (The Psychiatric Genomics Consortium, 2014). Rare risk alleles in the form of CNVs have also been identified; these typically confer relatively high risk of disorder (odds ratios 2–60) and in total occur in about 3% of cases as inherited or de novo mutations (Giusti‐Rodríguez & Sullivan, 2013). Whole‐exome sequencing studies support a polygenic contribution to the disorder from both inherited and de novo rare single nucleotide variants (SNVs) and insertion/deletion variants (Fromer et al., 2014; Genovese et al., 2016; Purcell et al., 2014). Studies documenting a burden of rare nonsynonymous SNVs in people with the disorder suggest that, as for GWAS and CNV analyses, the application of large samples will ultimately deliver significant findings for this class of risk variant (Zuk et al., 2014). Recent support for this comes from a recent meta‐analysis of 4,264 schizophrenia cases, 9,343 controls, and 1,077 parent‐proband trios in which genome‐wide significant support was obtained for rare loss‐of‐function SNVs in the gene *SETD1A* (Singh et al., 2016).

We have previously shown that alleles represented on exome arrays capture a fraction of the risk for schizophrenia attributable to rare SNVs (Richards et al., 2016) but, as with sequencing studies, our study was underpowered to implicate specific genes. To enhance power, we have increased the sample size to 10,011 schizophrenia cases and 13,791 controls by combining two of the largest schizophrenia case‐control cohorts available from the United Kingdom (5,585 cases and 8,103 controls) and Sweden (4,426 cases and 5,688 controls). The analysis of the UK sample exome chip data has previously been published (Richards et al., 2016), as has the Sweden whole exome sequencing results (though not the Sweden exome chip data) (Genovese et al., 2016).

We performed three primary analyses. These were single variant association using mixed model analysis, gene association using SKAT‐O, and gene set analysis using a burden test in SKAT. The candidate gene sets were chosen on the basis of available evidence from other types of genetic study of neuropsychiatric disorders (for more details see section 2.4). We hypothesized that, as the rarity of the variants and the large multiple testing correction was likely to lead to limited power to detect true associations for single variants (see section 2.5), the candidate gene sets with good prior evidence had the best chance of capturing a true association with schizophrenia.

## 2 METHODS AND MATERIALS

### 2.1 Samples

Sample sizes are given in Supplementary Table S1. The UK schizophrenia cases were from the CLOZUK and Cardiff COGS cohorts, both described previously and that are typical of schizophrenia with respect to the heritability conferred by common alleles (Hamshere et al., 2013). CLOZUK cases were prescribed the antipsychotic clozapine. This is primarily used for treatment‐resistant schizophrenia, so the CLOZUK cases are likely to be enriched for treatment resistance. In the United Kingdom, patients taking clozapine provide blood samples to allow the detection of adverse drug‐effects. Following ethical approval, we obtained anonymous blood samples (Hamshere et al., 2013). Cardiff COGS cases were recruited from community mental health teams in Wales and England on the basis of a clinical diagnosis of schizophrenia or schizo‐affective disorder (depressed sub‐type) as described previously (Carroll et al., 2011). After written informed consent, diagnosis was subsequently established using the Schedules for Clinical Assessment in Neuropsychiatry (SCAN) instrument (Wing et al., 1990) and review of case notes followed by consensus diagnosis according to DSM‐IV (American Psychiatric Association, 1994) criteria. Controls were taken from the UK Blood Service donors (4,455 samples) and the 1958 British Birth Cohort (4,615 samples) (Power & Elliott, 2006; Power et al., 2007; WTCCC, 2007). The study had UK Multicenter Research Ethics Committee approval.

Swedish cases with schizophrenia were identified via the Swedish Hospital Discharge Register which captures all public and private inpatient hospitalizations (Genovese et al., 2016; Ripke et al., 2013; Szatkiewicz et al., 2014). Cases were required to have two or more inpatient admissions for schizophrenia or schizo‐affective disorder. The validity of this case definition of schizophrenia is strongly supported (Dalman, Broms, Cullberg, & Allebeck, 2002; Kristjansson, Allebeck, & Börje, 1987). All procedures were approved by ethical committees at the Karolinska Institute in Sweden, and all subjects provided written informed consent (or legal guardian consent and subject assent). Controls were selected at random from Swedish population registers, and had never been hospitalized for schizophrenia, schizo‐affective disorder, or bipolar disorder.

For replication of rs61749465, we obtained data from an additional UK (UCL) schizophrenia cohort of 1,305 subjects who had received a clinical diagnosis of schizophrenia according to ICD‐10 which was subsequently confirmed by interviews using the Schedule for Affective Disorders and Schizophrenia—Lifetime edition (SADS‐L) (Endicott & Spitzer, 1978). The UCL control cohort included 1,309 subjects (480 were unscreened healthy UK subjects from the European Collection of Animal Cell Culture). The remaining UCL controls had no personal history of any RDC‐defined mental disorder and no family history of schizophrenia, alcohol dependence, or bipolar disorder. All cases and controls were of United Kingdom or Irish ancestry as described previously (Datta et al., 2010). UK National Health Service multicenter and local research ethics approvals were obtained and signed informed consent was given by all subjects.

*Genotyping* of the primary datasets was performed using Illumina HumanExome or HumanOmniExpressExome arrays (see URLs below). Whole exome sequencing of ∼10% of the Sweden cohort was used in array design. We restricted our analyses to the exome content contained in both arrays (*N* = 247,870 SNVs). Genotypes were called using Illumina GenomeStudio with subsequent processing of genotype with zCall (Goldstein et al., 2012) with batch‐specific intensity data. Cardiff COGS, CLOZUK, UK Blood Service donors, and the Swedish cohort were genotyped at the Broad Institute (Cambridge, MA). The 1958 British Birth Cohort was genotyped by the Wellcome Trust Sanger Institute.

Replication data for rs61749465 was genotyped in the UCL sample using a KASPar assay (LGC Genomics, Hoddesdon, UK) and heterozygotes confirmed by Sanger sequencing.

*Quality Control* (*QC*) was performed following the procedures we previously described (Richards et al., 2016). In brief, marker QC consisted of exclusions based on call rate <99%, Hardy–Weinberg Equilibrium (HWE) *p* < 1 × 10^−6^ in cases and controls separately, *p* < 5 × 10^−4^ in case/case batch comparisons, *p* < 1 × 10^−3^ in control/control batch comparisons and passing cluster plot separation checks (markers with GenTrain score < 0.4 or mean cluster separation < 0.08 were excluded). QC steps for subject exclusions were based on call rate <98%, relatedness based on identity by descent $\hat{\pi }$  < 0.1, heterozygosity, and PCA for population stratification. Of 6,991 cases and 9,070 controls initially available for the UK cohorts, 5,585 cases and 8,103 controls were retained. Principal component analysis (PCA) was used to control for population stratification. As in the previous analysis, CLOZUK/COGS PCA was performed using SmartPCA 3.0 on 5,128 variants with MAF > 0.01 and 1,100 samples from 11 populations using HapMap 3 (Thorisson, Smith, Krishnan, & Stein, 2005) as reference panel (Patterson, Price, & Reich, 2006; Price et al., 2006).

QC details for the Swedish cohort (4,610 cases and 5,894 controls before QC) are given in Supplementary Table S2. Marker QC consisted of exclusions based on call rate < 98% and HWE *p* < 1 × 10^−6^ in cases and controls separately. QC steps for subject exclusions were based on call rate <98%, relatedness based on identity by descent $\hat{\pi }$  < 0.1, heterozygosity, and PCA for population stratification. In the Swedish cohort, PCA was performed with SmartPCA v3.0 (Price et al., 2006) using LD pruned genome‐wide SNPs (these data were not available for all UK controls). Samples that were >6 standard deviations from the mean on PCA1 to PCA10 were dropped, and the process iterated 10 times. After QC, we retained 4,426 cases and 5,688 controls in the Swedish cohort. PLINK1.9 was used to perform all QC steps except for PCA (Purcell et al., 2007).

In total, there were 10,011 cases and 13,791 controls in the combined sample.

### 2.2 Allelic association

*Allelic association testing* was performed in GCTA (Yang, Lee, Goddard, & Visscher, 2011), using mixed linear model based association analysis (MLMA) based on the leave‐one‐chromosome‐out method (MLMA‐loco) (Yang, Zaitlen, Goddard, Visscher, & Price, 2014). This method provides controls for population stratification and sample relatedness. We concentrate on 112,950 variants with MAF < 0.01 (Supplementary Table S3).

### 2.3 Gene‐level association

We implemented tests to summarize the evidence for gene‐level association based on all nonsynonymous variants (MAF < 0.001, 92,815 variants) in a gene. This MAF threshold was chosen because it captured the greatest proportion of the rare variant signal in a exome‐sequencing study of schizophrenia (Fromer et al., 2014). We used SeqMeta 1.6.5 (URLs) available in R for meta‐analysis of the United Kingdom and Swedish cohorts to calculate unified Sequencing Kernel Association (SKAT‐O) tests and burden tests for genes. The burden test collapses minor alleles within a gene or pathway into a single variable (Li and Leal, 2008; Madsen & Browning, 2009) and is the most powerful approach when most of the minor alleles in the gene of pathway increase risk. SKAT aggregates genetic information by using multiple logistic regression in a kernel framework and is more powerful than the burden test when minor alleles show a mixture of risk or protective effects (Wu et al., 2011). The unified test (SKAT‐O) maximizes power by finding the optimal linear combination of the burden and SKAT approaches (Lee et al., 2012).

We annotated variants with MAF < 0.001 to genes according to the RefSeq hg19 (URLs). For gene‐wide association tests, we included only genes containing ≥2 variant sites in the datasets (13,443 genes; Supplementary Table S4). For both cohorts, we included 11 covariates (10 ancestry principal components and a covariate for genotyping platform).

### 2.4 Gene‐set analyses

Gene‐sets were selected given a priori evidence of enrichment for rare alleles. We thus conducted only the burden test using the SeqMeta package in R. As schizophrenia is highly polygenic, gene‐sets analyses are at their most informative when they are competitive against the genomic background (de Leeuw, Neale, Heskes, & Posthuma, 2016) so we included a covariate corresponding to the rare allele count for each individual for variants outside the candidate pathways.

We defined candidate gene sets based on previous evidence of enrichment for rare alleles from sequencing studies of schizophrenia (Tables 3 and S5; Supplementary Material for more information on how these pathways are derived) (Akawi et al., 2015; Bragin et al., 2014; Chen & Dent, 2014; Chiurazzi, Schwartz, Gecz, & Neri, 2008; de Ligt et al., 2012; De Rubeis et al., 2014; Lek et al., 2015; Fromer et al., 2014; Giusti‐Rodríguez & Sullivan, 2013; Khare et al., 2012; Network and Pathway Analysis Subgroup of Psychiatric Genomics Consortium, 2015; Najmabadi et al., 2011; Purcell et al., 2014; Rauch et al., 2012; Singh et al., 2016; The Psychiatric Genomics Consortium, 2014; van Bokhoven, 2011; Yun, Wu, Workman, & Li, 2011). For generic pathway exploration, we extracted 8,737 pathways from six publically available repositories (Supplementary Tables S6 and S7 and Supplementary Material) (Ashburner et al., 2000; Croft et al., 2014; Eppig et al., 2015; Gene Ontology Consortium, 2015; Kanehisa & Goto, 2000; Kanehisa et al., 2014; Mi, Muruganujan, Casagrande, & Thomas, 2013; Mi, Muruganujan, & Thomas, 2013; Milacic et al., 2012; Schaefer et al., 2009). We performed gene‐set analyses based on the full set of exonic variants (Supplementary Table S6), and another set of analyses restricted to damaging mutations (those annotated as “stop” or “splice”; Supplementary Table S7).

### 2.5 Statistical power

**Figure 1:**
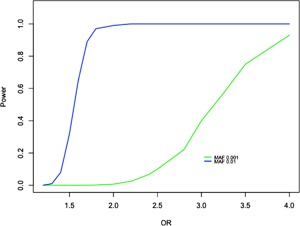
Power calculations for SNVs under an additive allelic model. Power calculations assume a variant with MAF = 0.001 (green line) or 0.01 (blue line) and a sample size of 10,011 cases and 13,791 controls. Significance is set at *α* = 1.2 × 10^−7^. Similar results are obtained for a dominant model given the low MAF

Given our sample sizes, we had 95% power to detect association to an allele with a MAF of 0.001 and odds ratio >4 at an exome‐wide significance threshold (*p* < 1.2 × 10^−7^, as suggested for moderate impact nonsynonymous variants; Sveinbjornsson et al., 2016). Statistical power was <1% to detect alleles at this frequency with an OR of 2 (Figure 1).

## 3 RESULTS

### 3.1 Allelic association

No single variants reached the exome‐wide significance threshold (*p* < 1.2 × 10^−7^). One variant, rs61749465 (exm679123, in *MCPH1*), neared this level of significance (*p* = 3.8 × 10^−7^). However, we did not obtain replication evidence for rs61749465 (Fisher's exact test *p* = 0.12) for this allele in the sample from UCL (1,305 cases and 1,309 controls), nor did meta‐analysis of rs61749465 in the UCL sample with the UK and Swedish cohorts provide additional support (Fisher's combined probability test; *p* = 8.2 × 10^−7^). The results for variants with *p* < 1 × 10^−4^ are presented in Table 1 and results for all the variants in Supplementary Table S3. Overall, 12 variants showed evidence for association below 10^−4^, similar to the number expected under the null.

**Table 1:** SNV association tests

Variant	Chr	Position	A1	A2	MAF (cases)	MAF (controls)	Odds ratio	*p*	Gene
exm679123	8	6272353	G	A	0.00460	0.00199	1.196	3.78E‐07	MCPH1
exm237695	2	166003301	T	C	0.00180	0.00054	1.300	9.55E‐06	SCN3A
exm1212971	16	4253250	T	C	0.00055	0.00004	1.683	1.23E‐05	SRL
exm1511038	19	56539847	A	G	0.00055	0.00000	1.747	1.72E‐05	NLRP5
exm1217358	16	11001377	C	G	0.00000	0.00058	0.646	3.12E‐05	CIITA
exm750535	9	36147794	G	A	0.00090	0.00018	1.433	3.91E‐05	GLIPR2
exm843062	10	95400694	A	C	0.00574	0.00845	0.911	5.13E‐05	PDE6C
exm1330168	17	43308023	G	A	0.00015	0.00098	0.730	7.13E‐05	FMNL1
exm491315	5	142593652	C	T	0.00674	0.00906	0.917	7.40E‐05	ARHGAP26
exm1055306	13	20224268	G	T	0.00020	0.00094	0.743	7.67E‐05	MPHOSPH8
exm252299	2	189932953	A	G	0.00140	0.00065	1.278	8.85E‐05	COL5A2
exm888875	11	8478972	G	C	0.00045	0.00152	0.761	9.42E‐05	STK33

SNV association test results, limited to *p* < 1 × 10^−4^ and MAF < 0.01. Variant column denotes Exome chip probe ID. “Chr” column gives chromosome. Chromosome and position are according to human genome build 37. A1 and A2 are the alleles for each variant. Odds ratio is for the A1 allele.

### 3.2 Gene association tests

None of the gene association results exceeded the gene‐wide significance threshold for SKAT‐O or burden tests (*p* < 2.5 × 10^−6^, Bonferroni correction for 20,000 genes). Genes significant at *p* < 3 × 10^−3^ are given in Table 2. The complete list of gene level results is given in Supplementary Table S4.

**Table 2:** Gene‐wise tests

Gene	Chr	Gene start	Gene end	SKAT‐O *p*	Burden test *p*	Odds ratio (burden)	*N* SNVs
POLR1E	9	37485931	37503694	9.80E‐05	9.88E‐05	3.118	4
CEP192	18	12991360	13125051	9.91E‐05	6.02E‐01	1.066	23
ARHGEF28	5	72921982	73237818	1.38E‐04	8.36E‐05	1.634	21
DNAH11	7	21582832	21941186	1.82E‐04	9.87E‐05	1.369	66
FOCAD	9	20658307	20995954	2.58E‐04	2.70E‐01	1.193	24
CSDE1	1	115259533	115300671	2.98E‐04	5.13E‐01	0.836	8
WDR89	14	64063756	64108641	4.19E‐04	4.38E‐04	3.255	2
MYCL	1	40361095	40367687	5.31E‐04	4.93E‐02	0.538	3
MRGPRF	11	68771861	68780850	5.87E‐04	4.89E‐04	3.768	5
SETX	9	135136826	135230372	6.13E‐04	8.35E‐02	1.284	23
ZNF610	19	52839497	52870376	8.13E‐04	1.23E‐01	1.516	6

SKAT‐O and burden tests results (*p* < 0.001) for SNVs with MAF < 0.001. “Chr” column gives chromosome. Positions are for human genome build 37. SKAT‐O *p* denotes SKAT‐O gene association *p*‐value. Burden test *p* is the burden test gene association *p*‐value. Odds ratios are given for the burden tests. *N* SNVs is number of variants tested.

### 3.3 Pathway analyses

For the candidate gene set analysis using a burden test on rare variants (Table 3), two gene sets, FMRP targets (Fromer et al., 2014; Giusti‐Rodríguez & Sullivan, 2013) and those that are loss‐of‐function intolerant (defined as those with *p*Lin ≥ 0.9 (Lek et al., 2015; Genovese et al., 2016), were significantly enriched, each passing the Bonferroni threshold for this analysis of *p* < 4.1 × 10^−3^. Our exploratory analysis of public repositories of gene‐set annotations identified no additional gene set that passed the Bonferroni significant threshold level of 5.7 × 10^−6^ (for the 8,737 pathways) when all we tested all mutations or only those predicted to be loss‐of‐function (Supplementary Tables S6 and S7).

**Table 3:** Gene‐set tests

Candidate pathway	Burden *p* (all)	Odds ratio (all)	Standard error (all)	*N* SNVs (all)	*N* genes (all)	Burden *p* (LoF)	*N* SNVs (LoF)	Odds ratio (LoF)
ASD de novo nonsynonymous	0.075	1.007	0.004	24,153	2,698	0.934	1,680	0.999
ASD de novo loss of function	0.637	0.991	0.019	1,379	960	0.602	1,372	0.990
ARC/NMDAR	0.740	0.987	0.040	296	58	0.383	23	1.141
Calcium channels	0.980	0.999	0.047	194	28	0.143	17	0.673
Developmental delay	0.048	1.013	0.006	10,013	1,284	0.635	719	0.986
FMRP targets	0.003	1.023	0.008	7,022	810	0.978	351	0.999
Histones	0.070	1.034	0.019	1,201	188	0.585	73	0.956
Loss of function intolerant	0.003	1.014	0.005	16,831	2,808	0.213	829	0.969
PGC2 SZ genome‐wide significant	0.166	1.023	0.016	1,614	295	0.034	110	1.165
Post synaptic density (PSD)	0.671	0.995	0.011	3,389	602	0.602	198	1.027
Schizophrenia de novo nonsynonymous	0.633	1.003	0.007	8,661	922	0.741	561	0.990
Schizophrenia de novo loss of function	0.362	0.970	0.034	458	335	0.343	457	0.969

The results of burden test (Burden *p*) analyses of candidate gene sets limited to SNVs with MAF < 0.001. Tests involving all nonsynonymous variants or those that are loss of function are in columns labeled, respectively, all and LoF. *N* SNVs is the number of variants in pathway that pass quality control with MAF < 0.001. *N* genes (all) is number of genes in the pathway that contain at least one nonsynonymous variant. Burden *p* is for the burden test of association based on minor alleles.

## 4 DISCUSSION

Exome sequencing and CNV studies have demonstrated that very rare variants that confer substantial effects on risk make a contribution to the genetic architecture of schizophrenia. Postulating that a proportion of this architecture could be captured at low cost using Illumina exome arrays containing by uncommon genetic variation, we have conducted the largest rare‐variant study of schizophrenia to date.

We found no evidence supporting association to any variant present on Illumina exome arrays. Our high power to detect uncommon alleles (0.01 ≤ MAF ≥ 0.001) that confer a large effect (OR > 4) effectively excludes the possibility that such alleles are present on these arrays (Figure 1). The Sweden sample should have been particularly tractable to this approach given that exon variation from Sweden informed Illumina exome array design. These findings also constrain expectations of what might be delivered by larger studies based on these arrays. Our study does not, however, exclude the possibility that some of the alleles within this frequency range confer weaker effects on risk; indeed the gene‐set analyses (see section 3.3 and discussion below) imply that at least some do.

SKAT‐O and burden analyses designed to enhance power in the event of allelic heterogeneity also failed to implicate any single gene for schizophrenia. Two results here are notable. First, the association evidence for *WDR88* (*p* = 0.003) which we previously reported to be associated with schizophrenia (Richards et al., 2016), was considerably diminished by addition of the Swedish data, suggesting the previous report is likely to be a false positive. We similarly found no support for *SETD1A* (Supplementary Table S4) which was recently found to be significantly enriched for ultra‐rare loss‐of‐function mutations in people with schizophrenia (Singh et al., 2016). However, given that the association evidence in that study derived from extremely rare events and de novo loss‐of‐function mutations, none of which is represented on these Illumina exome arrays, our study should not be viewed as inconsistent with the earlier study.

Although we found no significant association signals for individual alleles or genes, we did find evidence that uncommon nonsynonymous mutations were weakly enriched in two gene sets, predicted targets of FMRP and genes that are intolerant of loss‐of‐function mutations. FMRP targets have been shown to be enriched in schizophrenia for exonic mutations (both de novo [Fromer et al., 2014] and segregating [Purcell et al., 2014]) while LoF intolerant genes have been shown to be enriched for rare exonic mutations in a large sequencing study of the disorder (Genovese et al., 2016). Both gene‐sets were also significantly enriched for common variation in the largest GWAS of schizophrenia (Pardiñas, 2016). Our findings in FMRP and LoF‐intolerant gene‐sets are, therefore, consistent with studies using a range of designs. The consistency of findings across markedly different types of genetic variation and in widely varying study designs is remarkable. It also provides a compelling body of evidence that identifying the causal genetic variation within these gene sets has the potential to provide true insights into the primary aetiology of schizophrenia.

The magnitude of the enrichments of these gene‐sets for mutations in the present study is much smaller (Table 3; ORs ≤ 1.023) than that reported in the most recent exome sequencing study (OR ≈ 1.2) (Genovese et al., 2016) but the latter was based on ultra‐rare variants (i.e., occurring once in the sample and not present in a large exome database; Lek et al., 2015). This class of mutation is expected to be more highly enriched for damaging mutations than those represented on exome arrays. Restricting our analyses to variants on the arrays that are predicted to be loss‐of‐function did not enhance the signal in these pathways (Table 3). The differences in the variant frequency profiles between arrays and sequencing may also explain the absence of signals in other gene sets that have been consistently implicated in the disorder through CNV and exome sequencing, particularly the smaller gene sets such as ARC and NMDAR (Fromer et al., 2014; Purcell et al., 2014).

In conclusion, in the largest exome study of schizophrenia to date, we fail to implicate individual risk alleles or risk genes. We confirm enrichments in two gene‐sets that have previously been strongly implicated in the disorder. The associations in these pathways arise from exonic variation that is rare (MAF < 0.001) but not ultra‐rare or uniquely present in a single person. Associations to individual alleles or genes within this pathway should be achievable using this technology, although the sample sizes required will have to be larger than those that brought the early successes in GWAS of the disorder.

## URLS

Exome SNP genotyping selection: http://genome.sph.umich.edu/wiki/Exome_Chip_Design. SeqMeta, https://github.com/DavisBrian/seqMeta. Gene list for hg19: https://www.cog-genomics.org/plink2/resources#genelist

## Supporting information

Additional Supporting Information may be found online in the supporting information tab for this article.

Supporting Information S1.

Supporting Table S1.
