# Supplementary material for: Mutation intolerant genes and targets of FMRP are enriched for nonsynonymous alleles in schizophrenia
Source: Am J Med Genet B Neuropsychiatr Genet. 2017 Jul 18;174(7):724–31. doi: 10.1002/ajmg.b.32560 (PMC5669020; doi:10.1002/ajmg.b.32560)
Supplement: Supplementary file 1 — Supporting Information S1. [file AJMG-174-724-s001.docx]

*Supplementary Material*

We defined candidate gene sets based on previous evidence that they may be enriched for rare alleles from sequencing studies of schizophrenia (Supplementary Table 5); voltage gated calcium channel genes(Giusti-Rodríguez and Sullivan, 2013), brain-expressed genes that interact with the Fragile X mental retardation (FMRP)(Giusti-Rodríguez and Sullivan, 2013; Fromer et al., 2014), members of the ARC and NMDAR complexes(Giusti-Rodríguez and Sullivan, 2013; Fromer et al., 2014), genes from the human post-synaptic density, and genes affected by *de novo* mutations in schizophrenia(Purcell et al., 2014; Fromer et al., 2014). Given evidence from sequencing studies that genes impacted by rare variation in schizophrenia overlap with those similarly impacted by rare variation in developmental delay and autism spectrum disorder (ASD), we included a gene-set constructed from genes affected by *de novo* mutations in ASD(Chiurazzi et al., 2008; van Bokhoven, 2011; Najmabadi et al., 2011; Rauch et al., 2012; de Ligt et al., 2012; Bragin et al., 2014; Akawi et al., 2015; De Rubeis et al., 2014). Given that the histone modification has been implicated in schizophrenia from rare variant(Singh et al., 2016) and GWAS data(Network and Pathway Analysis Subgroup of Psychiatric Genomics Consortium, 2015), and in ASD(De Rubeis et al., 2014), we tested candidate pathways related to histone alterations based on the Histome database(Khare et al., 2012) and from review papers(Yun et al., 2011; Chen and Dent, 2014). See Supplementary Table 5, for gene membership for all these pathways. We also included all genes mapping to the 105 autosomal loci associated with schizophrenia in the 2014 PGC report(The Psychiatric Genomics Consortium, 2014). We excluded the extended MHC region as its inclusion would be expected to adversely affect power due to the large number of genes in the associated region. Finally, we included 3230 constrained genes that are loss-of-function intolerant (pLi ≥ 0.9) as these have been shown to be enriched for rare mutations in sequencing studies of neurodevelopmental disorders including schizophrenia(Exome Aggregation Consortium, 2015; Genovese et al., 2016).

For generic pathway exploration, we extracted comprehensive sets from Gene Ontology, Kyoto Encyclopedia of Genes and Genomes, National Cancer Institute, Mouse Genome Informatics, Protein Analysis Through Evolutionary Relationships and Reactome repositories(Ashburner et al., 2000; Gene Ontology Consortium, 2015; Kanehisa et al., 2014; Kanehisa and Goto, 2000; Schaefer et al., 2009; Eppig et al., 2015; Mi et al., 2013b, 2013a; Croft et al., 2014; Milacic et al., 2012). The total list consisted of 8,737 pathways.

Supplementary references

Akawi, N, McRae, J, Ansari, M, Balasubramanian, M, Blyth, M, Brady, AF, Clayton, S, Cole, T, Deshpande, C, Fitzgerald, TW, Foulds, N, Francis, R, Gabriel, G, Gerety, SS, Goodship, J, Hobson, E, Jones, WD, Joss, S, King, D, Klena, N, Kumar, A, Lees, M, Lelliott, C, Lord, J, McMullan, D, Osio, D, Piombo, V, Prigmore, E, Rajan, D, Rosser, E, Sifrim, A, Smith, A, Swaminathan, GJ, Turnpenny, P, Whitworth, J, Wright, CF, Firth, H V, Barrett, JC, Lo, CW, FitzPatrick, DR, Hurles, ME. 2015. Discovery of four recessive developmental disorders using probabilistic genotype and phenotype matching among 4,125 families. Nat. Genet. 47: 1363–1369.

Ashburner, M, Ball, CA, Blake, JA, Botstein, D, Butler, H, Cherry, JM, Davis, AP, Dolinski, K, Dwight, SS, Eppig, JT, Harris, MA, Hill, DP, Issel-Tarver, L, Kasarskis, A, Lewis, S, Matese, JC, Richardson, JE, Ringwald, M, Rubin, GM, Sherlock, G. 2000. Gene Ontology: tool for the unification of biology. Nat. Genet. 25: 25–29.

van Bokhoven, H. 2011. Genetic and epigenetic networks in intellectual disabilities. Annu. Rev. Genet. 45: 81–104.

Bragin, E, Chatzimichali, EA, Wright, CF, Hurles, ME, Firth, H V., Bevan, AP, Swaminathan, GJ. 2014. DECIPHER: Database for the interpretation of phenotype-linked plausibly pathogenic sequence and copy-number variation. Nucleic Acids Res. 42.

Chen, T, Dent, SYR. 2014. Chromatin modifiers and remodellers: regulators of cellular differentiation. Nat. Rev. Genet. 15: 93–106.

Chiurazzi, P, Schwartz, CE, Gecz, J, Neri, G. 2008. XLMR genes: update 2007. Eur. J. Hum. Genet. 16: 422–434.

Croft, D, Mundo, AF, Haw, R, Milacic, M, Weiser, J, Wu, G, Caudy, M, Garapati, P, Gillespie, M, Kamdar, MR, Jassal, B, Jupe, S, Matthews, L, May, B, Palatnik, S, Rothfels, K, Shamovsky, V, Song, H, Williams, M, Birney, E, Hermjakob, H, Stein, L, D’Eustachio, P. 2014. The Reactome pathway knowledgebase. Nucleic Acids Res. 42.

Eppig, JT, Blake, JA, Bult, CJ, Kadin, JA, Richardson, JE, Anagnostopoulos, A, Babiuk, RP, Baldarelli, RM, Beal, JS, Bello, SM, Berghout, J, Blodgett, O, Butler, NE, Corbani, LE, Cousins, SL, Dene, H, Drabkin, HJ, Forthofer, KL, Hale, P, Hutchins, L, Knowlton, M, Law, M, Lewis, JR, McAndrews, M, Miers, DS, Montenko, H, Ni, L, Onda, H, Pittman, W, Recla, JM, Reed, DJ, Richards-Smith, B, Sitnikov, D, Smith, CL, Tomczuk, M, Washburn, LL, Zhu, Y. 2015. The Mouse Genome Database (MGD): Facilitating mouse as a model for human biology and disease. Nucleic Acids Res. 43: D726–D736.

Exome Aggregation Consortium. 2015. Analysis of protein-coding genetic variation in 60,706 humans 2. Hear. Lung: 1–26.

Fromer, M, Pocklington, AJ, Kavanagh, DH, Williams, HJ, Dwyer, S, Gormley, P, Georgieva, L, Rees, E, Palta, P, Ruderfer, DM, Carrera, N, Humphreys, I, Johnson, JS, Roussos, P, Barker, DD, Banks, E, Milanova, V, Grant, SG, Hannon, E, Rose, SA, Chambert, K, Mahajan, M, Scolnick, EM, Moran, JL, Kirov, G, Palotie, A, McCarroll, SA, Holmans, P, Sklar, P, Owen, MJ, Purcell, SM, O’Donovan, MC, O’Donovan, MC. 2014. De novo mutations in schizophrenia implicate synaptic networks. Nature 506: 179–84.

Gene Ontology Consortium. 2015. Gene Ontology Consortium: going forward. Nucleic Acids Res. 43: D1049-56.

Genovese, G, Fromer, M, Stahl, EA, Ruderfer, DM, Chambert, K, Landén, M, Moran, JL, Purcell, SM, Sklar, P, Sullivan, PF, Hultman, CM, McCarroll, SA. 2016. Increased burden of ultra-rare protein-altering variants among 4,877 individuals with schizophrenia. Nat. Neurosci. 19.

Giusti-Rodríguez, P, Sullivan, PF. 2013. The genomics of schizophrenia: Update and implications. J. Clin. Invest. 123: 4557–4563.

Kanehisa, M, Goto, S. 2000. Kyoto Encyclopedia of Genes and Genomes. Nucleic Acids Res. 28: 27–30.

Kanehisa, M, Goto, S, Sato, Y, Kawashima, M, Furumichi, M, Tanabe, M. 2014. Data, information, knowledge and principle: Back to metabolism in KEGG. Nucleic Acids Res. 42.

Khare, SP, Habib, F, Sharma, R, Gadewal, N, Gupta, S, Galande, S. 2012. HIstome - A relational knowledgebase of human histone proteins and histone modifying enzymes. Nucleic Acids Res. 40.

de Ligt, J, Willemsen, MH, van Bon, BWM, Kleefstra, T, Yntema, HG, Kroes, T, Vulto-van Silfhout, AT, Koolen, D a, de Vries, P, Gilissen, C, del Rosario, M, Hoischen, A, Scheffer, H, de Vries, BB a, Brunner, HG, Veltman, J a, Vissers, LELM. 2012. Diagnostic exome sequencing in persons with severe intellectual disability. N. Engl. J. Med. 367: 1921–9.

Mi, H, Muruganujan, A, Casagrande, JT, Thomas, PD. 2013a. Large-scale gene function analysis with the PANTHER classification system. Nat. Protoc. 8: 1551–66.

Mi, H, Muruganujan, A, Thomas, PD. 2013b. PANTHER in 2013: Modeling the evolution of gene function, and other gene attributes, in the context of phylogenetic trees. Nucleic Acids Res. 41.

Milacic, M, Haw, R, Rothfels, K, Wu, G, Croft, D, Hermjakob, H, D’Eustachio, P, Stein, L. 2012. Annotating cancer variants and anti-cancer therapeutics in Reactome. Cancers (Basel). 4: 1180–1211.

Najmabadi, H, Hu, H, Garshasbi, M, Zemojtel, T, Abedini, SS, Chen, W, Hosseini, M, Behjati, F, Haas, S, Jamali, P, Zecha, A, Mohseni, M, Püttmann, L, Vahid, LN, Jensen, C, Moheb, LA, Bienek, M, Larti, F, Mueller, I, Weissmann, R, Darvish, H, Wrogemann, K, Hadavi, V, Lipkowitz, B, Esmaeeli-Nieh, S, Wieczorek, D, Kariminejad, R, Firouzabadi, SG, Cohen, M, Fattahi, Z, Rost, I, Mojahedi, F, Hertzberg, C, Dehghan, A, Rajab, A, Banavandi, MJS, Hoffer, J, Falah, M, Musante, L, Kalscheuer, V, Ullmann, R, Kuss, AW, Tzschach, A, Kahrizi, K, Ropers, HH. 2011. Deep sequencing reveals 50 novel genes for recessive cognitive disorders. Nature 478: 57–63.

Network and Pathway Analysis Subgroup of Psychiatric Genomics Consortium, TN and PAS of the PG. 2015. Psychiatric genome-wide association study analyses implicate neuronal, immune and histone pathways. Nat. Neurosci. 18: 199–209.

Purcell, SM, Moran, JL, Fromer, M, Ruderfer, D, Solovieff, N, Roussos, P, O’Dushlaine, C, Chambert, K, Bergen, SE, Kähler, A, Duncan, L, Stahl, E, Genovese, G, Fernández, E, Collins, MO, Komiyama, NH, Choudhary, JS, Magnusson, PKE, Banks, E, Shakir, K, Garimella, K, Fennell, T, DePristo, M, Grant, SGN, Haggarty, SJ, Gabriel, S, Scolnick, EM, Lander, ES, Hultman, CM, Sullivan, PF, McCarroll, S a, Sklar, P. 2014. A polygenic burden of rare disruptive mutations in schizophrenia. Nature 506: 185–90.

Rauch, A, Wieczorek, D, Graf, E, Wieland, T, Endele, S, Schwarzmayr, T, Albrecht, B, Bartholdi, D, Beygo, J, Di Donato, N, Dufke, A, Cremer, K, Hempel, M, Horn, D, Hoyer, J, Joset, P, R??pke, A, Moog, U, Riess, A, Thiel, CT, Tzschach, A, Wiesener, A, Wohlleber, E, Zweier, C, Ekici, AB, Zink, AM, Rump, A, Meisinger, C, Grallert, H, Sticht, H, Schenck, A, Engels, H, Rappold, G, Schr??ck, E, Wieacker, P, Riess, O, Meitinger, T, Reis, A, Strom, TM. 2012. Range of genetic mutations associated with severe non-syndromic sporadic intellectual disability: An exome sequencing study. Lancet 380: 1674–1682.

De Rubeis, S, He, X, Goldberg, AP, Poultney, CS, Samocha, K, Ercument Cicek, A, Kou, Y, Liu, L, Fromer, M, Walker, S, Singh, T, Klei, L, Kosmicki, J, Fu, S-C, Aleksic, B, Biscaldi, M, Bolton, PF, Brownfeld, JM, Cai, J, Campbell, NG, Carracedo, A, Chahrour, MH, Chiocchetti, AG, Coon, H, Crawford, EL, Crooks, L, Curran, SR, Dawson, G, Duketis, E, Fernandez, BA, Gallagher, L, Geller, E, Guter, SJ, Sean Hill, R, Ionita-Laza, I, Jimenez Gonzalez, P, Kilpinen, H, Klauck, SM, Kolevzon, A, Lee, I, Lei, J, Lehtimäki, T, Lin, C-F, Ma’ayan, A, Marshall, CR, McInnes, AL, Neale, B, Owen, MJ, Ozaki, N, Parellada, M, Parr, JR, Purcell, S, Puura, K, Rajagopalan, D, Rehnström, K, Reichenberg, A, Sabo, A, Sachse, M, Sanders, SJ, Schafer, C, Schulte-Rüther, M, Skuse, D, Stevens, C, Szatmari, P, Tammimies, K, Valladares, O, Voran, A, Wang, L-S, Weiss, LA, Jeremy Willsey, A, Yu, TW, Yuen, RKC, Cook, EH, Freitag, CM, Gill, M, Hultman, CM, Lehner, T, Palotie, A, Schellenberg, GD, Sklar, P, State, MW, Sutcliffe, JS, Walsh, CA, Scherer, SW, Zwick, ME, Barrett, JC, Cutler, DJ, Roeder, K, Devlin, B, Daly, MJ, Buxbaum, JD. 2014. Synaptic, transcriptional and chromatin genes disrupted in autism. Nature 515: 209–15.

Schaefer, CF, Anthony, K, Krupa, S, Buchoff, J, Day, M, Hannay, T, Buetow, KH. 2009. PID: The pathway interaction database. Nucleic Acids Res. 37.

Singh, T, Kurki, MI, Curtis, D, Purcell, SM, Crooks, L, McRae, J, Suvisaari, J, Chheda, H, Blackwood, D, Breen, G, Pietiläinen, O, Gerety, SS, Ayub, M, Blyth, M, Cole, T, Collier, D, Coomber, EL, Craddock, N, Daly, MJ, Danesh, J, DiForti, M, Foster, A, Freimer, NB, Geschwind, D, Johnstone, M, Joss, S, Kirov, G, Körkkö, J, Kuismin, O, Holmans, P, Hultman, CM, Iyegbe, C, Lönnqvist, J, Männikkö, M, McCarroll, SA, McGuffin, P, McIntosh, AM, McQuillin, A, Moilanen, JS, Moore, C, Murray, RM, Newbury-Ecob, R, Ouwehand, W, Paunio, T, Prigmore, E, Rees, E, Roberts, D, Sambrook, J, Sklar, P, Clair, DS, Veijola, J, Walters, JTR, Williams, H, Sullivan, PF, Hurles, ME, O’Donovan, MC, Palotie, A, Owen, MJ, Barrett, JC. 2016. Rare loss-of-function variants in SETD1A are associated with schizophrenia and developmental disorders. Nat. Neurosci. 19: 571–577.

The Psychiatric Genomics Consortium. 2014. Biological insights from 108 schizophrenia-associated genetic loci. Nature 511: 421–427.

Yun, M, Wu, J, Workman, JL, Li, B. 2011. Readers of histone modifications. Cell Res. 21: 564–78.

Supplemental Tables

Supplemental Table 1. Genotyping platform, phenotype, genotyping centre, initial sample size and initial SNP number by data batch.

Supplemental Table 2. Variant and sample quality control criteria and thresholds for CLOZUK/COGS and Swedish datasets.

Supplemental Table 3. SNV association test results using mixed model analysis, limited to MAF<0.01. ‘Variant’ column shows Exome chip probe ID. ‘Variant rs ID’ shows rs ID. ‘Chromosome’ and ‘Position’ columns show chromosome and human genome build 37 positions. ‘A1’ and ‘A2’ show the alleles for each variant. Column ‘MAF’ shows the minor allele frequency across cases and controls, while ‘MAF (cases)’ and ‘MAF (controls)’ show MAF for cases and controls alone. ‘Odds ratio’ shows the odds ratio for the A1 allele. ‘Standard error’ and ‘P’ columns show standard error and P values for the mixed model association test. ‘Function’ shows the functional category of the variant (nonsynonymous, splice or stop). ‘Gene’ indicates which gene the variant is found in.

Supplemental Table 4. Gene SKAT-O and burden tests results for SNVs with MAF < 0.001. Positions shown are for human genome build 37. ‘Gene’ column shows gene symbol. ‘Chromosome’, ‘gene start’ and ‘gene end’ columns show chromosomal position (human genome build 37). ‘SKAT-O P’ shows SKAT-O gene association p-value. ‘Burden test P’ shows burden test gene association p-value. ‘Odds ratio (burden)’ shows odds ratio for burden association. ‘N SNVs’ shows number of variants used for test.

Supplemental Table 5. Gene membership for candidate gene-sets.

Supplemental Table 6. Burden tests of functional gene sets taken from GO, KEGG, NCI, MGI, PANTHER and Reactome repositories. Limited to SNVs with MAF < 0.001, for nonsynonymous variants. ‘Pathway’ column shows depository and pathway ID number. ‘Burden P’, ‘Odds ratio’ and ‘Standard error’ show the p-value, odds ratio and standard error for the burden test association for all nonsynonymous variants. ‘N SNVs (all)’ shows number of nonsynonymous variants in pathway that pass quality control and have MAF < 0.001. ‘Description’ shows the function of the genes in the pathway.

Supplemental Table 7. Burden tests of functional gene sets taken from GO, KEGG, NCI, MGI, PANTHER and Reactome repositories. Limited to SNVs with MAF < 0.001, for loss of function variants only. ‘Pathway’ column shows depository and pathway ID number. ‘Burden P’, ‘Odds ratio’ and ‘Standard error’ show the p-value, odds ratio and standard error for the burden test association for all nonsynonymous variants. ‘N SNVs (all)’ shows number of nonsynonymous variants in pathway that pass quality control and have MAF < 0.001. ‘Description’ shows the function of the genes in the pathway.
